# Pragmatic randomised controlled trial to evaluate the effectiveness and cost effectiveness of a multi-component intervention to reduce substance use and risk-taking behaviour in adolescents involved in the criminal justice system: A trial protocol (RISKIT-CJS)

**DOI:** 10.1186/s12889-017-4170-6

**Published:** 2017-03-11

**Authors:** Simon Coulton, Kelly Stockdale, Catherine Marchand, Nadine Hendrie, Jenny Billings, Sadie Boniface, Steve Butler, Paolo Deluca, Colin Drummond, Dorothy Newbury-Birch, Tracy Pellatt-Higgins, Alex Stevens, Alex Sutherland, Ed Wilson

**Affiliations:** 1 Centre for Health Services Studies, University of Kent, Canterbury, UK; 2 Health and Social Care Institute, Teesside University, Middlesbrough, UK; 3 Institute of Psychiatry Psychology and Neurosciences, Kings College, London, UK; 4 Addaction Young Peoples Service, Canterbury, Kent UK; 5 School of Sociology, Social Policy and Social Research, University of Kent, Canterbury, Kent UK; 6 RAND Europe, Cambridge, UK; 7 Dept. of Public Health and Primary Care, University of Cambridge, Cambridge, UK

**Keywords:** Adolescent, Youth offending, Criminal justice, Substance use, Risk-taking behaviour, Randomised controlled trial, Health economics, Motivational interviewing

## Abstract

**Background:**

Adolescence is a critical developmental stage when young people make lifestyle choices that have the potential to impact on their current and future health and social wellbeing. The relationship between substance use and criminal activity is complex but there is clear evidence that the prevalence of problematic substance use is far higher among adolescent offenders than the general adolescent population. Adolescent offenders are a marginalized and vulnerable population who are significantly more likely to experience health and social inequalities in later life than their non-offending peers.

There is a paucity of evidence on effective interventions to address substance use and risk-taking behaviours in adolescent offender populations but it is clear that preventative or abstinence orientated interventions are not effective. RISKIT-CJS is an intervention developed in collaboration with young people taking account of the current best evidence. Feasibility and pilot studies have found the intervention addresses the needs of adolescents, is acceptable and has demonstrated potential in reducing substance use and risk-taking behavior.

**Methods:**

The study is a mixed method, two-armed, prospective, pragmatic randomized controlled trial with individual randomisation to either treatment as usual alone or the RISKIT-CJS intervention in addition to treatment as usual. Adolescents, aged 13 to 17 years inclusive, engaged with the criminal justice system who are identified as having problematic substance use are eligible to participate. The study will be conducted across three geographical areas; South and South East England, London and North East England between March 2017 and February 2019.

**Discussion:**

The study represents an ambitious programme of work to address an area of need for a marginalized and vulnerable population.

**Trial registration:**

ISRCTN77037777 registered 15/09/2016.

## Background

Adolescence is a critical developmental stage when young people make behavioural and lifestyle choices that have the potential to impact on their current, and future, health and wellbeing. While risk-taking is important for healthy psychological development, for many, inappropriate risk-taking, often in the form of substance use such as alcohol, illicit substances, legal highs and inappropriate use of prescription medication, is associated with health and social harm that can persist well into adulthood [1]. Adolescents are far more vulnerable to the negative impact of substance use due to a range of psychological factors that often interact and the differential effect of substances on the developing brain [2].

The relationship between substance use and criminal activity is complex but it is clear that the prevalence of substance use is higher in adolescent offending populations and the two are related in the context of other forms of disinhibitory behavior such as aggression and risk-taking [3]. Adolescents who offend experience a range of complex risks and vulnerabilities including neglect and abuse [4], substance use related problems, and exclusion from education [5, 6]. As a group, they are more likely to experience health and social inequalities in later life, such as poor physical health [7], early pregnancy in females [8], higher rates of drug and alcohol dependence [5, 6, 9], reduced employment opportunities and economic hardship [10]. There is a widespread consensus that adolescents who offend are one of the most vulnerable and ‘hard to reach’ groups in the United Kingdom [11], which has one of the highest youth custody populations in Western Europe [12]. In common with other vulnerable populations adolescents in the criminal justice system are more likely to access physical and mental health services in times of crisis and this access is often mediated through other agencies so identification of those in need and early intervention strategies should be proactive rather than reactive [9, 13, 14].

Recent data indicates that 14% of annual arrests in England and Wales involved adolescents aged between 10 and 17 years, equating to 296 000 arrests. In 2012/13 there were almost 28 000 first time adolescent offenders and the rate of recidivism in this population is high at 36% [15]. The prevalence of problematic substance use is also high with 32% experiencing problems associated with their use and 12% experiencing severe problems [16].

The development of the RISKIT intervention [17], and subsequently RISKIT-CJS, was based on two streams of work; a thorough review of the research evidence and a participative consultation with young people. The theoretical perspective was informed by the Social Development Model (SDM) [18–20]. This approach suggests that the distal influences of socio-economic status, biology, normative regulation and discipline are mediated through proximal influences on behaviour which are identified as; perceived opportunities for pro- or antisocial behaviour and perceived rewards for this behaviour. The SDM marries the ecological context of young people’s behaviour to an explanation of how this ecology influences their behaviour. It suggests that even in the absence of a structural change to their health ecology, the provision of socio-emotional and cognitive skills can help young people prevent or reduce risk-taking behaviour and also suggests that the building of bonds with organisations promoting pro-social learning and opportunities is important in the reduction of risk-taking. The model provides a coherent and empirically validated approach that suggests that intervention approaches should be multi-component and encompass; knowledge and education, cognitive and learning skills, self-efficacy and motivation.

The participatory consultation was adapted from participatory action research [21, 22] and was carried out with a number of groups of young people. The aim of the exercise was to establish, with young people what they perceived as risk-taking behaviour, why they took risks, the consequences of taking risks and how they perceived the problems could be addressed. The main themes in terms of risk-taking behaviour centred around; criminal activity, substance use and sexual activity and these activities were considered as being linked. The participants considered prevention programmes, that focus on the negative outcomes of risk, failed to appreciate that risk-taking can be positive and lead to positive outcomes, an issue highlighted by other research exploring the processes associated with risk-taking [18, 23]. The young people highlighted the need for education regarding risks and consequences associated with substance use, but particularly highlighted the preference for interventions that provided skills and strategies to manage risk and the opportunity to discuss these skills with peers and to learn how to implement them. Interestingly parental influences were not considered critical to any intervention and many considered parental involvement would be inappropriate and unacceptable. The primary focus for the young people was not on eradication of risk-taking but rather a focus on how substance use and risk-taking could be reduced and the negative consequences minimised.

We consulted a number of existing reviews and research studies [24–29] and found that while there is a growing body of research in the field there is a paucity of rigorously evaluated interventions with the majority of research arising from the US with limited applicability to the UK. Of importance was what has been proven not to work, this includes focusing on negative aspects of risk and risk abstinence. Promising intervention approaches included motivational interviewing and cognitive and socio-emotional life skills training. In addition there was emerging recognition of the importance of providing interventions in a structured manner and with the young people’s preference for peer group interventions the importance of managing the potentially negative effects of labelling and peer influence.

Synthesis of the participatory group views, theoretical underpinnings and the review of the evidence was undertaken and the original RISKIT intervention model developed as an approach that focuses on those who are vulnerable to the negative consequences of their risk-taking behaviour. The intervention combines individual motivational interviewing sessions, to target motivation and behaviour change with eight one hour group orientated life skills sessions that covered a variety of areas; identifying and managing risk, communication skills, assertiveness training, anger management, preparing for behaviour change, sexual health. In addition the group sessions focused on identifying resources within the community that could be of benefit for the young people and provided opportunities to access these resources.

An initial feasibility study was undertaken followed by a larger pilot study [17] in adolescents identified as engaging in excessive risk-taking behaviour. Consent rates in the eligible population were high, 80%, with almost all attending at least part of the intervention and 74% attending all of the intervention sessions. Follow-up rates were also high with 82% being followed-up at 6 months. Overall 32% of the intervention group had reduced their risk-taking behaviour to a point where it was of no further concern and the impact of the intervention led to a greater reduction in substance use than the control condition indicating a positive effect on this domain. Participant views were positive with high levels of engagement and satisfaction and a general view that the intervention had been useful in developing new skills, informative and had led to changes in behaviour. Delivery of the model was sustainable but required the input of specialist, rather than generic staff and a full economic evaluation of cost-effectiveness was not undertaken. Further to our pilot study the RISKIT intervention has been modified for delivery in custodial and community criminal justice populations, RISKIT-CJS. Assessment of feasibility demonstrated high levels of satisfaction on the part of the participants. Consent and engagement was high with 90% consenting and almost 100% attending, in part because the group intervention was provided over two four-hour sessions over consecutive weeks, on weekends, rather than the 8 weekly one-hour sessions provided in the pilot study.

The proposed study builds on the Medical Research Council guidelines for the development and evaluation of complex interventions. We have conducted research to explore the theoretical validity of the intervention and synthesized this theoretical approach with the current evidence base and the views of potential participants in order to model an appropriate intervention approach. We have tested the feasibility of implementing the intervention in the target population and refined the intervention and its delivery as a result of that feasibility study. We have conducted an appropriately designed pilot study to explore potential effectiveness on the key parameters and found evidence of potential effect in reducing substance use and risk-taking behaviour and high levels of satisfaction and engagement. As the proposal involves a specific population, those engaged with the criminal justice system rather than adolescents per se, we have conducted a second feasibility study in this population to assess feasibility and acceptability and found high levels of engagement and acceptability in this population. The next step is to conduct a rigorous evaluation to address key outcomes in a way that provides valid scientific evidence and is useful to those engaged with this population and commissioners of services. To this end we have proposed a full, multi-centre randomized controlled trial, with an embedded qualitative component, of the intervention versus treatment as usual to explore the effectiveness of the intervention; in reducing substance use and risk-taking behaviours, improving mental-wellbeing, and reducing criminal activity that is acceptable to participants and economically viable to deliver.

### Aims of the study

To evaluate the effectiveness and cost-effectiveness of the RISKIT-CJS intervention compared with treatment as usual in reducing substance use and related harms for adolescents engaged with the criminal justice system.

### Objectives

- To conduct a pragmatic prospective randomized controlled trial to evaluate the effectiveness of the RISKIT-CJS intervention compared with treatment as usual for substance using adolescents involved in the criminal justice system.
- To evaluate the cost-effectiveness of the intervention compared with treatment as usual.
- To explore participants, practitioners and criminal justice staff experience, and acceptability, of the intervention.
- To assess the fidelity of the intervention delivery and explore the role of fidelity, therapeutic alliance and baseline psychological factors on any outcomes observed.

## Methods and design

**Fig. 1:**
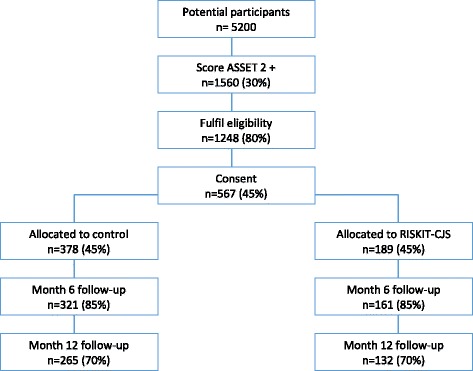
Trial Flow diagram

The trial has been granted ethical approval by the University of Kent Ethics Committee (Ref: SRCEA169) and will be conducted in accordance with the Declaration of Helsinki between March 2017 and February 2019. A Full flow diagram for the study is provided in Fig. 1.

### Design

A mixed method, two-arm, prospective, pragmatic randomized controlled trial with individual allocation. Randomisation will be to either treatment as usual alone or the RISKIT-CJS intervention in addition to treatment as usual. The study will be conducted across three geographical areas; South and South East England, London and North East England.

#### Study hypotheses

The primary and secondary hypotheses, stated as null hypotheses are;

- The RISKIT-CJS intervention is no more effective than treatment as usual at 12-months post-randomisation in reducing the frequency of substance use in the previous 28-days.
- The RISKIT-CJS intervention is no more cost-effective than treatment as usual at 12-months post-randomisation.

### Setting

Twenty-four Youth Offending Teams (YOT) across England will be recruited (North-East *n* = 8, South-East *n* = 8, London and the South *n* = 8). The sample is designed to reflect variation in terms of social deprivation and affluence, rural and urban, and culturally mixed populations.

### Participants

#### Inclusion criteria

Aged between 13 and 17 years inclusive, engaged with a participating YOT, scoring 2 or more on the ASSET, or equivalent, assessment for substance use.

#### Exclusion criteria

Severity of substance use requiring immediate referral to specialist services for detoxification, known criminal justice involvement likely to lead to incarceration during the intervention or follow-up period, currently on an order with substance use abstinence as a pre-requisite.

### Randomisation

Randomisation will be conducted at the level of the participant and by an independent, secure trial unit using random permuted blocks of variable length. To allow for the most efficient use of resources differential allocation will be employed with twice as many participants allocated to the control group compared with the intervention group. Power calculations have been adjusted to reflect the allocation ratio. Randomisation will include stratification by gender, YOT and age group (13–15 years versus 16–17 years).

### Sample size

The effectiveness analysis is designed to identify a clinically important difference of 0.3 for the primary outcome measure, percent days abstinent from all substances at 12-months post-randomisation. In order to detect an effect size difference of 0.3, with alpha of 0.05 and 80% power using a two-sided test requires 175 analysed at 12 months in each of the two groups, a total of 350. As the intervention is intensive and potentially costly to implement we have increased the efficiency of the study by allocating participants in a 2:1 ratio, with twice as many allocated to the control condition. As differential allocation leads to a loss of power we have maintained the integrity of the sample size calculation by increasing the numbers required to maintain power at 80%; 264 in the control group and 132 in the intervention group, a total of 396. Our previous studies with adolescents and those involved in the criminal justice system [30] suggests that loss at 12 months is likely to be somewhere in the region of 15 to 30% and we have adjusted the required sample to account for a 30% loss at 12 months. This inflates the required number consenting to 567; 378 in the control group and 189 in the intervention group. The optimal intervention group size is 8 participants and we aim to deliver 24 RISKIT-CJS groups, 8 in each of the geographical areas.

The qualitative component of the study will be purposive and include group discussion with participants in the intervention group and individual interviews with staff in participating YOT’s. Participants will be chosen purposively in order to provide diversity in terms of site, and age and ensure appropriate participation by gender, social class and ethnicity. The sample size considerations of the qualitative component are driven by the need to achieve data saturation, the point at which no new themes are emerging from the data, and this needs to be judged in practice. Our previous experience of similar studies would estimate the numbers groups of participants to be somewhere of the order of 12 groups and the number of staff in YOT to be of the order of 24.

### Interventions

#### RISKIT-CJS

RISKIT-CJS is delivered in 4 steps consisting of two one-to-one sessions lasting approximately one hour each and two half-day group sessions over two consecutive weeks. Groups are delivered in mutually convenient premises by trained and experienced practitioners in the delivery of therapeutic interventions to young people, all of whom are provided with training in the RISKIT-CJS intervention and ongoing supervision and support. The optimal group size is 6–8 participants.

The intervention involves four distinct steps. Step 1 entails a single 60-min face-to-face session using motivational interviewing approaches to explore current substance use, risk-taking behaviour, and support for behaviour change and enhance motivation to engage with the intervention. This session is provided immediately after randomisation.

Step 2 involves a group session over half a day at a location convenient for the participants. This session uses a group CBT approach and addresses a number of key issues involving both psycho-education and skill development including; understanding substance use and associated harms, understanding triggers of substance use behaviour, strategies for managing and minimizing risk-taking behaviour, strategies for diversion and distraction and sexual health concerns.

Step 3 is conducted a week later at the same location as step 2 and involves a similar group approach. At this session issues covered include communication strategies and assertiveness training, managing anger, using mindfulness and planning for the future.

Step 4 is a single one-to-one session using a motivational interviewing approach that addresses outstanding barriers to change, managing expectancy and enhancing self-efficacy to change. At this stage interventionists work with participants to identify local service contacts that may be useful.

#### Training practitioners to deliver RISKIT-CJS

Experienced youth workers with prior training and accreditation in providing motivational interviewing will be trained using an existing training programme. The training is provided over 2 days and covers; theoretical underpinnings, delivering programme elements, managing groups, individual motivational interviews, managing risks and safeguarding. A full training and practice manual is available for practitioners and senior practitioners will observe practice and deem practitioners as competent prior to embarking on the RISKIT-CJS programme. Supervision is provided by senior staff with experience of delivering the intervention on a regular basis throughout the study period.

### Control

Guidelines suggest that interventions should be provided for adolescents within CJS settings who score 2 or more on ASSET for substance use, but the reality is that there exists no standard intervention approach with those who have no immediate clinical need for treatment, in the form of detoxification or substitution. More often existing services are signposted and rates of engagement are low [14]. When intervention is provided it often takes the form of short duration brief behavioural change interventions with limited evidence of effectiveness in adolescent substance using populations [31].

### Outcome measures

#### Screening for substance use

ASSET is a standardized assessment tool, developed within the criminal justice system in England and Wales, which aims to identify the underlying causes of a young person’s offending behaviour and to plan appropriate interventions [32]. It is often used on multiple occasions to help measure changes in young offenders’ health and social needs and the risk of reoffending over time. ASSET has been used with all young offenders in England and Wales since 2000 and it examines 12 dynamic risk factors; living arrangements, family and personal relationships, education, neighbourhood, lifestyle; substance use, physical health, emotional health, perception of self and others, thinking and behaviour, attitudes to offending, and motivation to change. The severity of each section is rated on a 0–4 scale [32]. A score of 2 or more on the substance use domain of ASSETT is indicative of substance use related problems that are associated with offending activity.

#### Primary outcome measure

Our primary outcome measure is percent days abstinent from substance use in the 28-days prior to the 12-month follow-up. This is measured using the Time-Line Follow Back 28 day (TLFB28), a valid and reliable tool for assessing the quantity and frequency of substance use over time periods ranging from 1 to 365 days. The outcome has been validated for use in adolescent populations [33] and recent pilot work has indicated high levels of agreement between the shorter, 28-day, and longer 90-day, reference period. In addition to percent days abstinent the tool allows derivation of a number of secondary outcomes over the period; quantity and type of substances consumed, sexual activity (planned, unplanned and regretted) and incidences of self-harming behaviour. The TLFB is completed by a trained member of research staff and takes approximately 20 min. The outcome is measured at baseline, 6 and 12 months.

#### Secondary outcome measures

Mental health and wellbeing will be assessed using the Warwick-Edinburgh Mental Well-being scale (WEMWBS). WEMWBS is a 14-item, self-completed questionnaire addressing different aspects of eudemonic and hedonic mental health wellbeing. The scale has established valid reliable psychometric properties in adolescent populations [34] and established sensitivity to change [35], the instrument is highly correlated with other measures of psychological health and well-being [36]. WEMWBS will be measured at baseline and then again at 6 and 12 months.

The Strengths and Difficulties Questionnaire (SDQ) [37] is a brief, 25-item, self-completed questionnaire designed to explore common emotional and behavioural difficulties in adolescent populations. In addition, the questionnaire identifies the presence and severity of a number of common mental health disorders in accordance with ICD-10 criteria; conduct-oppositional, inattention, anxiety-depression [38]. The questionnaire has established psychometric properties and performs well when compared with other, more extensive tools. SDQ will be assessed at baseline and then again at 6 and 12 months.

In order to assess potential prognostic factors, in addition to demographics, that may impact on outcome we will assess motivation to change, measured using the Readiness to change questionnaire, (RCQ-TV) [39], and self-efficacy, measured using the Brief Situational Confidence Questionnaire, (BSCQ) [40] at baseline. Both instruments are relatively short self-completed questionnaires with established psychometric properties in the adolescent population.

#### Economic outcome measures

The economic outcome measures will address the costs of delivering the interventions, any change in health utility in the 12 months after randomization and the service costs associated with participants in the 12 months after randomization. Costs associated with delivering the intervention will be derived using a micro-costing approach accounting for the actual costs including associated training, facilities, overheads and management costs. Health utility will be assessed using the self-completed 5-item EQ-5D-5 L assessed at baseline, 6 and 12 months. Service utilization on the part of the participant will be assessed using a specifically designed client receipt service inventory (CRSI) [41], currently being piloted with the adolescent population. Service use will be assessed from a wide public sector perspective encompassing health and social care; criminal justice, education and employment service utilisation.

Criminal justice outcomes will include arrests, charges and convictions and will be derived from both Police National Computer Systems and YOT Management Information Systems. Data will be collected for all offence types for the 12 months prior to and 12 months after randomization.

#### Process outcome measures

The process of delivering the intervention may also play a role in the outcomes observed in the intervention group and we aim to assess this process using two distinct approaches. First, at the end each of the one-to-one sessions we will ask each participant to complete the Therapeutic Alliance Scale for Children (TASC-r; [42]) a 12-item self-completed instrument with established psychometric properties in the adolescent population. Second a random sample of 20% of individual motivational interviewing interventions, stratified by centre, age and gender, will be recorded and assessed by independent raters using the Behavioural Change Counselling Index (BECCI; [43]) to assess fidelity and quality of interventions delivered.

### Procedure

#### Assessment of potential eligibility

All adolescents within YOT are routinely screened using the ASSET tool or an equivalent and the results of the screen are held on secure electronic records. Members of the study team will liaise with YOT staff to identify potential participants who meet the substance use eligibility criteria and arrange a meeting at the YOT office between potential participants and the RISKIT-CJS practitioner, where possible this meeting will be scheduled to coincide with existing commitments on the part of the participant.

#### Consent

Practitioners will meet with participants at the YOT office. Eligibility will be assessed and those eligible provided with a written and verbal description of the study and invited to consider participating. If a participant is willing to participate consent will be taken for those aged 16 or more or those considered by the YOT staff as being ‘Gillick’ competent. For those not considered competent to provide consent caregivers will be contacted, provide with information about the study and asked to provide informed consent. Potential participants, whether consenting or not, will be provided with a £10 voucher to compensate them for their time.

#### Follow up assessment

Follow up will be conducted at 6 months and 12-months post randomization and researchers will be blind to participant allocation. Two weeks prior to the 6 and 12-month follow-up assessment participants will be contacted by phone and post to make an appointment to carry out the follow-up assessment with the option to complete the assessment by phone if no suitable location can be identified. The 6 and 12-month assessment will be similar to the baseline assessment and all participants will be provided with a £10 voucher to compensate for their time and to reduce attrition in the follow-up sample [44].

#### Qualitative data collection

Twelve RISKIT-CJS groups will be purposefully selected according to geographical region and group dynamics, and a group discussion will be conducted at the end of step 3 of the intervention, the last group session attended. The group discussion will be facilitated by an individual with experience of participating in the RISKIT-CJS intervention and observed by an experienced qualitative researcher. The approach employed will be Participatory Rapid Appraisal [45] to elicit in depth exploration of the acceptability and perceived effectiveness of the program and of the different elements within it.

In order to explore the RISKIT-CJS intervention from the perspective of the practitioners, six semi-structured phone interviews will be conducted, 2 weeks after the final motivational interviewing intervention. The semi-structured interviews and field notes maintained by interventionists will be used to explore a number of key objectives; feasibility, acceptability and perceived effectiveness of the programme.

Telephone interviews will be carried out with a purposively selected sample of YOT staff who are involved with RISKIT-CJS programme, chosen according to profession and region. The aim is to explore the impact of the RISKIT-CJS intervention from the perspective of the YOT staff who work with the target population. There will be 24 semi-structured telephone interviews with staff across the participating teams. The Interviews will be conducted 4 weeks after the final step four of the motivational interviewing intervention.

## Analysis

### Effectiveness analysis

Effectiveness analysis will be conducted by treatment allocation using a two-sided 5% significance level. Analysis and results will be presented in accordance with CONSORT guidelines. The primary outcome is percent days abstinent from substance use in the 28-days prior to the 12-month follow-up. After checking for distributional assumptions and making any appropriate transformations, this will be analysed using an analysis of covariance adjusting for baseline values and stratification values used in the randomisation process; age, gender and YOT. Results will be presented as mean differences between the groups and the associated 95% confidence intervals. Missing data will be assessed using multiple imputation approaches to model missing data scenarios, and sensitivity analysis will be conducted to explore the relative impact of missing data on the observed outcome. Secondary outcomes will be modelled in a similar manner.

Analysis will also be undertaken to model the relationship between pre-randomisation factors and observed outcomes at 12-months; demographics, self-efficacy, readiness to change. This analysis will employ a linear regression model including interaction terms for randomised group. To further enhance our understanding, we will additionally incorporate an analysis of process by enhancing the prognostic analysis through the inclusion of measures of adherence, fidelity derived from the BECCI ratings, and therapeutic alliance.

Exploratory sub-group analysis will be undertaken to model the relationship between gender, ethnicity and socio-economic status, measured using an index of material deprivation from postcode on observed outcome.

### Cost-effectiveness analysis

The effectiveness analysis will be complemented by an economic evaluation that will evaluate the economic implications of the intervention versus treatment as usual. Substance use generates high costs for the individual, health service and society in general and the economic analysis will be conducted first using a narrow health and social care perspective, to concord with National Institute for Health and Social Care Excellence guidelines for the conduct of health economic evaluations; and second using a wider public sector perspective incorporating costs associated with employment, training, education and criminal justice.

The costs associated with identifying the eligible population and delivering the interventions will be estimated by prospectively monitoring local costs associated with this activity and micro-costing training, management, supervision, facilities and associated overheads. Data will be extracted from study billing records. Impact on service use on the part of the participant will be estimated using a specifically designed CSRI and units of service use valued using national unit costs.

The economic analysis will comprise cost-consequences analysis (CCA) and incremental cost-effectiveness (CEA) and cost-utility analyses (CUA). The CCA will report mean and confidence intervals of: primary and secondary outcomes as described above as well as QALYs gained, costs per participant in each arm to health and social services, the criminal justice system and society as a whole, and differences between arms. The CEA will comprise calculation of the incremental cost per incremental day free of substance use, and the CUA, incremental cost per QALY gained. QALYs will be calculated from EQ-5D-5 L data converted to health state utilities using preference weights specific to the UK population and integrated over time. All analyses will be conducted over the time horizon of 1 year. Analysis of uncertainty will comprise reporting of standard errors and 95% confidence intervals around increments and calculation of the cost-effectiveness acceptability curve. Non-parametric bootstrapping will be employed to investigate joint uncertainty in costs and effects and both one-way and multi-way sensitivity analysis will be conducted to explore the impact of our basic assumptions. The study will be conducted and reported in accordance with good practice guidelines for health economic evaluations [46].

### Criminal justice outcomes

Criminal justice outcomes will be analysed using a combination of linear and logistic analysis of covariance to explore changes between groups and associated differences. A secondary analysis of CJS data will incorporate baseline risk assessments to explore the potential intervention effects on participant risk status.

### Qualitative analysis

The aim of the qualitative component of the proposal is to explore participants’ and practitioners’ experience of the RISKIT-CJS intervention in order to generate information pertaining to the feasibility, acceptability, contextual influences and mechanisms of action. The qualitative design consists of two elements; the first is participatory group work with participants who have experienced the RISKIT-CJS programme and the second telephone interviews with practitioners who work with these participants. Thematic coding of qualitative data will be carried out using specific software (QSR NVIVO) that allows for the coding of both verbal, transcripts and field notes, and visual data. The coding allows for the identification of recurrent and important themes and the generation of a framework of themes relating to the key research questions and objectives. A detailed description of themes will be presented. Emergent themes that may be explored from the quantitative data analysis will be incorporated into the analysis plan as secondary exploratory analyses.

## Discussion

There is a paucity of current research evidence regarding effective interventions to address substance use, risk-taking and related problems for adolescents engaged in the criminal justice system. The current study builds on a body of work that has developed a promising intervention and piloted it with success in adolescent populations. The proposed study represents an innovative and ambitious programme of work evaluating the RISKIT-CJS intervention in reducing substance use in this population. The study will inform theory and practice within and beyond the UK and provide important information on the effectiveness, cost effectiveness and implementation of interventions to address a recognized area of need in a vulnerable and hard to reach population.

## Abbreviations

BECCI: Behavioural change counselling index
BSCQ: Brief situational confidence questionnaire
CBT: Cognitive behavioural therapy
CCA: Cost consequence analysis
CEA: Cost-effectiveness analysis
CJS: Criminal justice system
CRSI: Client receipt service inventory
CUA: Cost-utility analysis
EQ-5D-5 L: Euroqual 5 dimension 5 level questionnaire
ICD-10: International classification of disease 10th edition
NHS: National Health service
NICE: National institute of clinical excellence
NIHR: National institute for health research
QALY: Quality adjusted life year
RCQ-TV: Readiness to change – treatment version questionnaire
SDM: Social development model
SDQ: Strengths and difficulties questionnaire
TASC-R: Therapeutic alliance scale for children- revised
TLFB28: Time line follow back 28 day
WEMWEBS: Warwick Edinburgh mental well-being scale
YOT: Youth offending team

## References

[1] Odgers CL (2008). Is it important to prevent early exposure to drugs and alcohol among adolescents?. Psychol Sci.

[2] Copeland WE (2013). Diagnostic transitions from childhood to adolescence to early adulthood. J Child Psychol Psychiatry.

[3] Newbury-Birch D (2015). Alcohol-related risk and harm amongst young offenders aged 11–17. Int J Prison Health.

[4] Social Exclusion Unit (2002). Reducing reoffending by ex-prisoners.

[5] Galahad SMS Ltd (2009). Evaluation of the Substance Misuse Project in the Young Person’s Secure Estate.

[6] Galahad SMS Ltd (2004). Substance Misuse and Juvenile Offenders.

[7] Coffey C (2003). Mortality in young offenders: retrospective cohort study. BMJ.

[8] Ritakallio M (2005). Delinquent behaviour and depression in middle adolescence: A Finnish community sample. J Adolesc.

[9] Bardone A (1998). Adult physical health outcomes of adolescent girls with conduct disorder, depression and anxiety. J Am Acad Child Adolesc Psychiatry.

[10] Willmott D, Van Olphen J (2005). Challenging the health impacts of incarceration: The role for community health workers. Californian Journal of Health Promotion.

[11] British Medical Association (2014). Young lives behind bars: the health and human rights of children and young people detained in the criminal justice system.

[12] Khan L (2010). Reaching out, reaching in: Promoting mental health and emotional well-being in secure settings.

[13] Anderson L, Vostanis P, Spencer N (2004). Health needs of young offenders. J Child Health Care.

[14] Stallard P, Thomason J, Churchyard S (2003). The mental health of young people attending a Youth Offending Team: a descriptive study. J Adolesc.

[15] Youth Justice Board and Ministry of Justice (2014). Youth Justice Statistics 2012/13. England and Wales.

[16] Wilson E (2013). Youth Justice Interventions - Findings from the Juvenile Cohort Study (JCS).

[17] Stevens A (2014). RISKIT: The participatory development and observational evaluation of a multi-component programme for adolescent risk-behaviour reduction. Drugs: Educ, prev & Pol.

[18] Catalano R, Hawking J, Hawkings J (1996). The Social Development Model: a theory of antisocial behaviour. Delinquency and crime: current theories.

[19] Catalano R (1999). A test of the social development model to predict problem behaviour during the elementary school period. Crim Beh & Men Health.

[20] Duerden M, Giullard A (2011). An approach to theory-based youth programming. New Directions for Youth Development.

[21] Cahill C (2007). Doing research with young people:Participatory research and the rituals of collective work. Children’s Geographies.

[22] Cornwall A, Jewkes R (1995). What is participatory research. Soc Sci & Med.

[23] Fagan J, Piquero AR (2007). Rational choice and developmental influences on recidivism among adolescent felony offenders. J Empir Leg Stud.

[24] Buhler A, Kroger C (2008). *Prevention of substance misuse*. *EMCDDA Insights*.

[25] Foxcroft DR (2002). Primary prevention for alcohol misuse in young people. Cochrane Database Syst Rev.

[26] Grimshaw GM, Stanton A (2006). Tobacco cessation interventions for young people. Cochrane Database Syst Rev.

[27] Kavanagh J (2006). A systematic review of the evidence for incentive schemes to encourage positive health and other social behaviours in young people.

[28] McGraph Y (2006). Review of the grey literature on drug prevention among young people.

[29] NICE (2007). Interventions to reduce substance use among vulnerable young people.

[30] Newbury-Birch D (2014). Alcohol screening and brief interventions for offenders in the probation setting (SIPS Trial): a pragmatic multicentre cluster randomized controlled trial. Alcohol Alcohol.

[31] Carney T, Myers B (2012). Effectiveness of early interventions for substance-using adolescents: findings from a systematic review and meta-analysis. Subst Abuse Treat Prev Policy.

[32] Youth Justice Board (2006). Asset - Young Offender Assessment Profile.

[33] Levy S (2004). Test-retest reliability of adolescents’ self-report of substance use. Alcohol Clin Exp Res.

[34] Clarke A (2011). Warwick-Edinburgh Mental Well-being Scale (WEMWBS): validated for teenage school students in England and Scotland. A mixed methods assessment. BMC Public Health.

[35] Maheswaran H (2012). Evaluating the responsiveness of the Warwick Edinburgh Mental Well-Being Scale (WEMWBS): group and individual level analysis. Health Qual Life Outcomes.

[36] Tennant R (2007). The Warwick-Edinburgh Mental Well-being Scale (WEMWBS): development and UK validation. Health Qual Life Outcomes.

[37] Goodman R (1997). The Strengths and Difficulties Questionnaire: a research note. J Child Psychol Psychiatry.

[38] Goodman R, Renfrew D, Mullick M (2000). Predicting type of psychiatric disorder from Strengths and Difficulties Questionnaire (SDQ) scores in child mental health clinics in London and Dhaka. Eur Child Adolesc Psychiatry.

[39] Heather N (1999). The developmentof a treatment version of the Readiness to Change Questionnaire. Addict Res.

[40] Breslin F (1998). A comparison of brief and long versions of the Situational Confidence Questionnaire. Beh Res & Ther.

[41] Deluca P (2015). Linked randomised controlled trials of face-to-face and electronic brief intervention methods to prevent alcohol related harm in young people aged 14–17 years presenting to Emergency Departments (SIPS junior). BMC Public Health.

[42] Sheik S, Saiz C (1992). Clinical, empirical and developmental; perspectives on the therapeutic relationship in child psychotherapy. Dev Psychopath.

[43] Lane C (2005). Measuring adaptions of motivational interviewing: the development and validation of the behaviour change counselling index (BECCI). Pat Educ & Couns.

[44] Willie N (2013). Systematic review of strategies to reduce attrition in randomised controlled trials.

[45] Beebe J (1995). Basic concepts and techniques of rapid appraisal. Human Org.

[46] Husereau D (2013). Consolidated Health Economic Evaluation Reporting Standards (CHEERS) statement. Int J Technol Assess Health Care.

